# Deciphering the Therapeutic Role of Lactate in Combating Disuse-Induced Muscle Atrophy: An NMR-Based Metabolomic Study in Mice

**DOI:** 10.3390/molecules29102216

**Published:** 2024-05-09

**Authors:** Yu Zhou, Xi Liu, Zhen Qi, Longhe Yang, Caihua Huang, Donghai Lin

**Affiliations:** 1 Key Laboratory of Chemical Biology of Fujian Province, Department of Chemical Biology, College of Chemistry and Chemical Engineering, Xiamen University, Xiamen 361021, China; zhouyu_921@163.com (Y.Z.); lancyliu2584@163.com (X.L.);; 2 Technical Innovation Center for Utilization of Marine Biological Resources, Third Institute of Oceanography, Ministry of Natural Resources, Xiamen 361021, China; 3 Research and Communication Center of Exercise and Health, Xiamen University of Technology, Xiamen 361021, China; huangcaihua@xmut.edu.com

**Keywords:** disuse-induced muscle atrophy, lactate, NMR spectroscopy, metabolomics, metabolism

## Abstract

Disuse muscle atrophy (DMA) is a significant healthcare challenge characterized by progressive loss of muscle mass and function resulting from prolonged inactivity. The development of effective strategies for muscle recovery is essential. In this study, we established a DMA mouse model through hindlimb suspension to evaluate the therapeutic potential of lactate in alleviating the detrimental effects on the gastrocnemius muscle. Using NMR-based metabolomic analysis, we investigated the metabolic changes in DMA-injured gastrocnemius muscles compared to controls and evaluated the beneficial effects of lactate treatment. Our results show that lactate significantly reduced muscle mass loss and improved muscle function by downregulating Murf1 expression, decreasing protein ubiquitination and hydrolysis, and increasing myosin heavy chain levels. Crucially, lactate corrected perturbations in four key metabolic pathways in the DMA gastrocnemius: the biosynthesis of phenylalanine, tyrosine, and tryptophan; phenylalanine metabolism; histidine metabolism; and arginine and proline metabolism. In addition to phenylalanine-related pathways, lactate also plays a role in regulating branched-chain amino acid metabolism and energy metabolism. Notably, lactate treatment normalized the levels of eight essential metabolites in DMA mice, underscoring its potential as a therapeutic agent against the consequences of prolonged inactivity and muscle wasting. This study not only advances our understanding of the therapeutic benefits of lactate but also provides a foundation for novel treatment approaches aimed at metabolic restoration and muscle recovery in conditions of muscle wasting.

## 1. Introduction

Disuse muscle atrophy (DMA) is a critical healthcare dilemma that manifests as the degradation and functional loss of muscle tissue following prolonged inactivity. This condition can result from a variety of clinical scenarios, including prolonged bed rest, fractures, amputations, or exposure to microgravity environments [1]. Recovery from DMA can be a long and challenging journey, especially for individuals who have experienced extended periods of immobilization or paralysis. This can lead to considerable muscle atrophy, decreased strength, and changes in metabolism. The paucity of effective treatments for DMA underscores the urgent need for innovative rehabilitation and preventative measures to counteract muscle degeneration and its subsequent consequences [2].

A comprehensive approach that combines specific physical exercise, especially resistance training, with a personalized nutritional plan has been identified as the most successful way to combat and potentially reverse muscle loss [3]. This integrated approach not only promotes muscle hypertrophy but also enhances muscle plasticity. Empirical evidence from studies of immobilization and bed rest in healthy subjects underscores the beneficial role of nutritional interventions, particularly those involving protein or amino acid supplementation, in averting muscle atrophy and preserving muscle functionality [4,5]. Furthermore, ongoing research indicates the potential of polyphenolic compounds in preventing muscle wasting by regulating protein degradation pathways, promoting protein synthesis, aiding in muscle regeneration, and enhancing mitochondrial efficiency [6]. While neuromuscular electrical stimulation has been proven to be effective in preserving muscle mass, its ability to maintain muscle strength is still limited, underscoring the importance of using it carefully to restore both muscle mass and function [7].

Lactate, a crucial component in energy metabolism, is typically linked to anaerobic conditions such as intense physical activity or oxygen scarcity. It is generated through the anaerobic degradation of glycogen within muscle cells [8]. Should muscle gluconeogenesis be insufficient, lactate will permeate the bloodstream through the cell membrane, eventually reaching the liver, where it is converted into glucose. This glucose is then reabsorbed by the muscles, completing the Cori cycle, a critical muscle–liver–muscle, glucose–lactate–glucose exchange that is vital during physical exertion and recovery [9]. Increasingly, lactate is being recognized as the principal circulating carbohydrate fuel that decouples mitochondrial energy production from glycolysis, offering new perspectives on the dynamics of energy metabolism [10].

In addition, the Cahill cycle, also known as the glucose–alanine cycle, parallels the function of the Cori cycle in its role during intense muscular activity. In this cycle, alanine produced from pyruvate and amino groups in muscle tissue is transported to the liver, where it is converted back to pyruvate and then to glucose, which is then returned to the muscle. This cycle plays a critical role in managing nitrogen waste and glucose levels during muscle recovery and catabolic states, further emphasizing the interconnected nature of muscle metabolism and systemic energy homeostasis [11,12].

Lactate not only serves as an essential energy source for the liver and other tissues but also significantly contributes to the acid–base homeostasis of the body and profoundly affects exercise performance [13,14]. Moreover, lactate is increasingly being recognized as a signaling molecule that can initiate mitochondrial adaptations, impacting the respiratory functions of mitochondria in the skeletal muscles of mice [15]. Recent studies have also broadened our comprehension of the biological relevance of lactate, underscoring its role in regulating diverse signaling pathways [16,17]. Functioning as a metabolic regulator, lactate stimulates the AMPK signaling pathway, enhances the proliferation and differentiation of myoblasts [18], and may suppress the cAMP pathway by activating GPR81 in a positive feedback mechanism. This fosters intracellular TG storage and mitochondrial stability in myotubes [19]. It is crucial to recognize the ongoing discussions and the necessity for further investigations into the role of lactate, influenced by factors such as concentration, tissue specificity, and pathological conditions. This reflects an ever-deepening understanding of its biological functions.

This study aimed to uncover the therapeutic efficacy of lactate in mitigating DMA. Preliminary observations indicate its potential to reduce muscle loss and improve muscle functionality in DMA-affected mice. Despite these encouraging findings, a thorough understanding of the metabolic complexities in DMA-impaired skeletal muscle and the molecular mechanisms by which lactate exerts its beneficial effects remains elusive [20,21].

Our study explored the metabolic landscape of the gastrocnemius muscle of DMA-affected mice, using NMR-based metabolomic profiling to assess the effects of lactate treatment. Our results provided profound insights into the metabolic changes induced by DMA and the restorative effects of lactate, particularly in terms of energy metabolism and amino acid metabolism. This study may not only broaden our understanding of the therapeutic potential of lactate in restoring metabolic balance and facilitating muscle recovery, but it may also pave the way for novel therapeutic strategies to combat muscle-wasting conditions.

## 2. Results

### 2.1. Lactate Reduced Gastrocnemius Muscle Mass Loss Induced by Hindlimb Suspension

**Figure 1:**
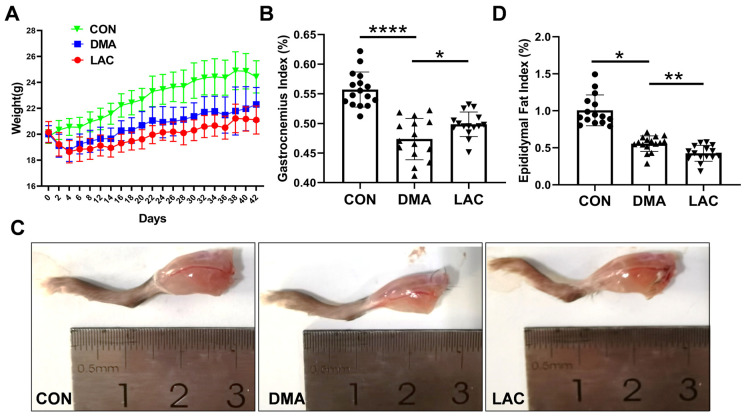
Effect of lactate treatment on phenotypes of the DMA mice. (**A**) Time course of body weight for the LAC, DMA, and CON mice from day 0 to day 42. (**B**) Gastrocnemius ratio: Ratio of gastrocnemius muscle wet weight to body weight. (**C**) Illustrative images of the right hindlimbs of mice. (**D**) Epididymal fat ratio: Ratio of epididymal fat weight to body weight. Data are presented as mean ± SD (*n* = 16). Levels of statistical significance: * *p* < 0.05; ** *p* < 0.01; **** *p* < 0.0001.

In the first phase of our study, we evaluated the effects of hindlimb suspension and subsequent lactate treatment on the overall body condition of mice. Our observations revealed that hindlimb suspension significantly hindered weight gain in DMA mice (the disuse-induced muscle atrophy group) compared to CON mice (the control group), with lactate treatment leading to a slight additional decrease in the body weight of LAC mice (the lactate-treated group) compared to DMA mice (Figure 1A). Hindlimb suspension also significantly reduced the ratio of gastrocnemius muscle wet weight to body weight. Significantly, lactate treatment attenuated the reduction in muscle mass induced by hindlimb suspension (Figure 1B,C). In addition, we examined the changes in epididymal fat content following suspension and lactate treatment (Figure 1D). Suspension resulted in a decrease in epididymal fat content, which was further reduced by lactate treatment, as reflected by the ratio of epididymal fat weight to body weight.

### 2.2. Lactate Improved Contractile Function in Gastrocnemius Muscles of Suspended Mice

**Figure 2:**
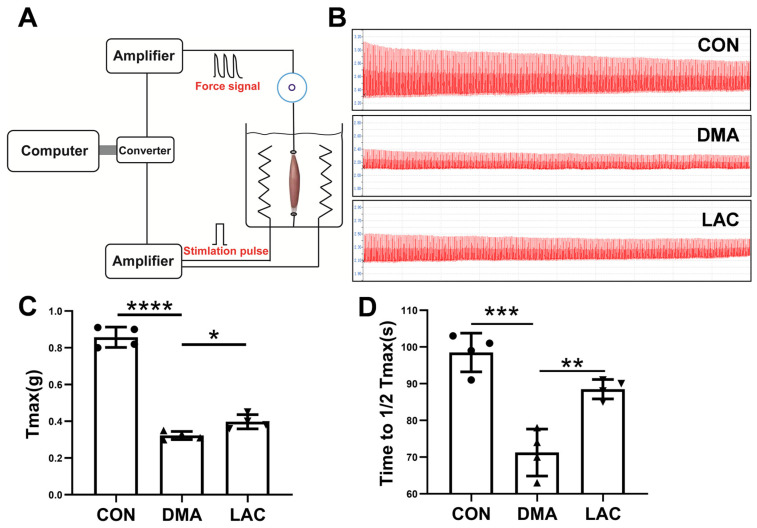
Effect of lactate treatment on the contractility and endurance of the gastrocnemius muscle in mice. (**A**) Schematic diagram of the setup for assessing ex vivo muscle contractility. (**B**) Typical force profile of the gastrocnemius muscle. (**C**) Peak contractile force (T_max_) of the muscle. (**D**) Endurance capacity, defined as the time required to reduce Tmax to 50% of its maximum value. Experimental data are presented as mean ± SD (*n* = 4). Statistical significance: *p* < 0.05, *; *p* < 0.01, **; *p* < 0.001, ***; *p* < 0.0001, ****.

Assessment of muscle contractility is critical for understanding muscle atrophy. To investigate the contractility of the gastrocnemius muscle, we performed in vitro experiments on isolated gastrocnemius muscles (Figure 2A). Our results showed that hindlimb suspension significantly impaired the maximal contractile force of the gastrocnemius muscle. However, treatment with lactate was effective in partially reversing this decrease in muscle force (Figure 2B,C). In addition, muscle endurance, the ability of muscles to sustain continuous effort, was significantly reduced after the suspension (Figure 2B,D). Lactate was beneficial in partially restoring this loss of muscle endurance. These results suggest that lactate treatment provides a protective benefit against the impairments in muscle contractility induced by hindlimb suspension.

### 2.3. Lactate Attenuated Muscle Atrophy in Suspended Mice

**Figure 3:**
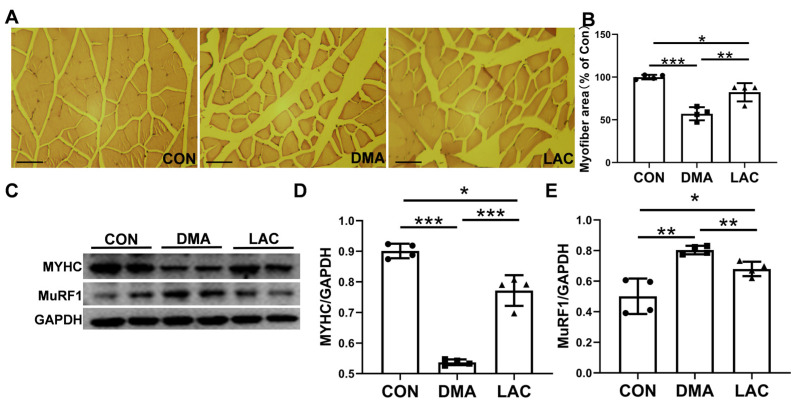
Effect of lactate treatment on the gastrocnemius muscle of hindlimb-suspended mice. (**A**) Pathomorphological analysis of cross-sectional hematoxylin and eosin (H&E) staining of the gastrocnemius muscle. Scale bar: 200 µm. (**B**) Quantitative analysis of muscle fiber cross-sectional area. (**C**–**E**) Western blot analysis (**C**) and densitometric quantification (**D**,**E**) to assess the expression levels of MuRF1 and MYHC in gastrocnemius muscle. Experimental data are presented as mean ± SD (*n* = 4). Statistical significance: *p* < 0.05, *; *p* < 0.01, **; *p* < 0.001, ***. GAPDH, glyceraldehyde 3-phosphate dehydrogenase.

Histologic studies were performed to investigate the effects of hindlimb suspension and lactate treatment on gastrocnemius muscle mass and contractility. Hematoxylin and eosin (H&E) staining revealed a significant decrease in muscle fiber cross-sectional area in the DMA group compared to the control group. In contrast, a significant increase was observed in the LAC group compared to the DMA group (Figure 3A,B). The expression of MuRF1 (muscle-specific RING finger protein 1), a key mediator of muscle atrophy, was significantly increased by suspension, a process that was significantly attenuated by lactate treatment (Figure 3C,D). In addition, the expression of MYHC (myosin heavy chain), which had decreased in atrophied muscles, was significantly increased after lactate treatment. These results suggest that lactate effectively counteracts muscle atrophy in hindlimb-suspended mice.

### 2.4. Lactate Altered the Metabolic Profile of Gastrocnemius Muscles in Suspended Mice

**Figure 4:**
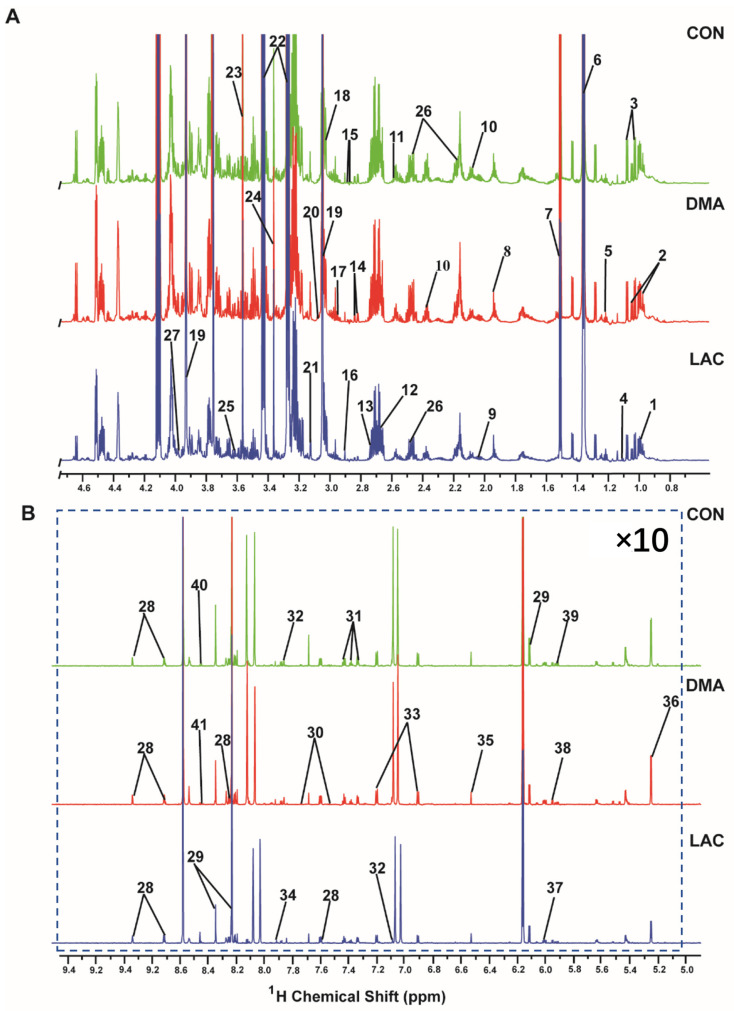
Two spectral regions are displayed: (**A**) 0.0–4.7 ppm; (**B**) 5.0–9.5 ppm. The spectra were recorded on a Bruker Avance III 850 MHz NMR spectrometer at 298 K (pH 7.4). Spectral regions of 0.0–4.7 ppm and 5.0–9.5 ppm are displayed, and the spectral region from 4.7 to 5.0 ppm was omitted to exclude the water peak. For enhanced clarity, the region from 5.0 to 9.5 ppm has been magnified 10 times relative to the region from 0.0 to 4.7 ppm. A total of 41 metabolites were identified: 1. Leucine; 2. Isoleucine; 3. Valine; 4. Methyl succinate; 5. Ethanol; 6. Lactate; 7. Alanine; 8. Acetate; 9. Proline; 10. Glutamate; 11. Citrate; 12. Carnosine; 13. Sarcosine; 14. Aspartate 15. Asparagine 16. Trimethylamine; 17. Glutathione; 18. Lysine; 19. Creatine phosphate; 20. Ornithine; 21. Malonate; 22. Taurine; 23. Glycine; 24. Methanol; 25. Threonine; 26. Glutamine; 27. Serine; 28. Niacinamide; 29. Inosine; 30. Tryptophan; 31. Phenylalanine; 32. Histidine; 33. Tyrosine; 34. Methyl histidine; 35. Fumarate; 36. Glucose; 37. UMP; 38. GTP; 39. Uridine; 40. NADH (nicotinamide adenine dinucleotide); 41. Formate.

Figure 4 shows the 1D ^1^H NMR spectra of aqueous extracts from the gastrocnemius muscles in the LAC, DMA, and CON groups of mice. A total of 41 metabolites were identified and their resonances were confirmed by 2D ^1^H-^13^C HSQC spectra (Figure S2 and Table S1). Excluding methanol due to the difficulty involved in distinguishing between its endogenous and exogenous origin in our samples, only 40 metabolites were included in the quantitative analysis of concentrations (Table S2). The range of identified metabolites includes twenty-three associated with amino acid metabolism and seven essentials for energy metabolism. In addition, the following metabolites were identified: the nucleotide UMP (uridylic acid); nucleosides such as uridine, GTP (guanosine triphosphate), and inosine; amine metabolites including trimethylamine and nicotinamide; and five carboxylic acid derivatives. These results significantly enhance our understanding of the metabolic changes caused by lactate in the context of DMA.

**Figure 5:**
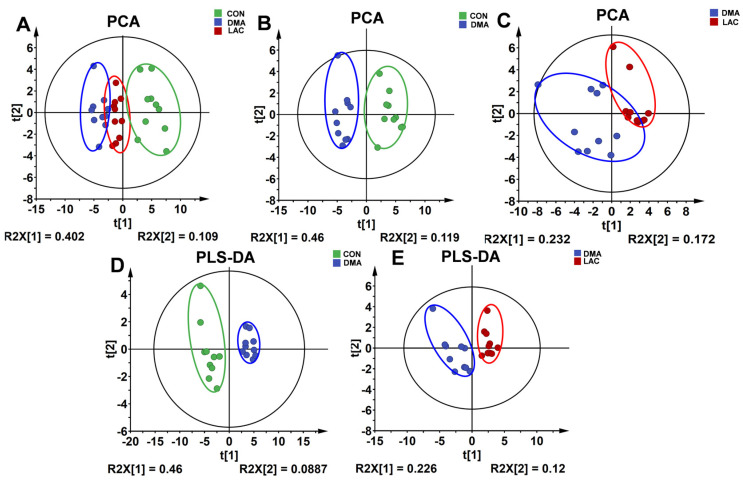
Score plots and cross-validation plots of the PCA and PLS-DA models using the NMR datasets from the three groups of mouse gastrocnemius. (**A**–**C**) PCA score plots provide an overview of the metabolic grouping among the three groups. (**D**,**E**) PLS-DA score plots provide an enhanced metabolic distinction between the DMA and CON groups (**D**) and the LAC and DMA groups (**E**).

To unravel the metabolic differences among the three gastrocnemius groups, we performed unsupervised PCA (principal component analysis) on the NMR datasets. The PCA score plots clearly show the unique metabolic profiles of each group. Specifically, the metabolic pattern of the LAC group was positioned intermediately between the other two groups along the first principal component (Figure 5A). In addition, the metabolic profile of the DMA group was different from that of the CON group (Figure 5B). Similarly, the metabolic profile of the LAC group was markedly different from that of the DMA group (Figure 5C).

We also performed supervised PLS-DA (partial least squares discriminant analysis) to enhance the differentiation between the three gastrocnemius groups. The PLS-DA score plots revealed significant changes in the metabolic profiles due to hindlimb suspension compared to controls (Figure 5D), and lactate treatment significantly altered the metabolic profile of the gastrocnemius in mice subjected to suspension (Figure 5E). In addition, a random permutation test (*n* = 200) was performed to verify the reliability of the two PLS-DA models. The cross-validation plots yielded the following metrics: R^2^Y (cum) = 0.959 and Q^2^Y (cum) = 0.898 for the comparison between the DMA and CON groups (Figure S3A), and R^2^Y (cum) = 0.893 and Q^2^Y (cum) = 0.631 for the comparison between the LAC and DMA groups (Figure S3B). These results underscore the high predictive accuracy and robustness of the PLS-DA models.

### 2.5. Identification of Characteristic Metabolites in Pairwise Comparisons between the Groups

Figure S4 presents a heat map illustrating the concentration patterns of metabolites within the LAC, DMA, and CON groups of mouse gastrocnemius muscle, revealing distinct trends in the metabolic changes. Notably, several metabolites in the DMA group showed concentration shifts that were inversely related to those in the CON group but closely matched the changes observed in the LAC group.

**Figure 6:**
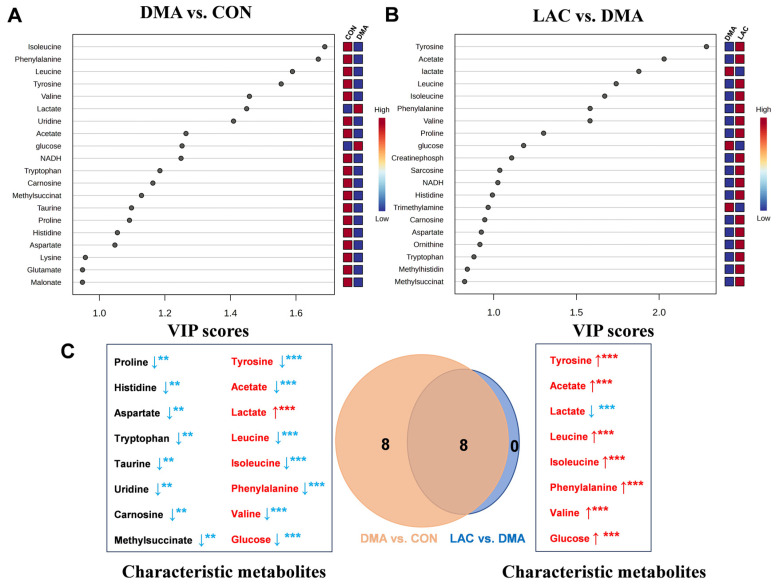
Significant metabolites and characteristic metabolites were identified from pairwise comparisons among three groups of mouse gastrocnemius. (**A**,**B**) VIP score ranking plots for significant metabolites derived from PLS-DA analysis of DMA vs. CON (**A**) and LAC vs. DMA (**B**). (**C**) Venn diagram showing characteristic metabolites from pairwise comparisons between DMA and CON and LAC and DMA groups, with common metabolites highlighted in red and ordered by decreasing VIP scores. Statistical significance: ** *p* < 0.01, *** *p* < 0.001. Red/blue indicates an increase/decrease in the relative metabolite levels, respectively.

Using a criterion of VIP > 1, we identified significant metabolites that differed between groups: 17 significant metabolites were identified between the DMA and CON groups (Figure 6A) and 12 between the LAC and DMA groups (Figure 6B). In addition, using a significance threshold of *p* < 0.05, we identified twenty-six differential metabolites between the DMA and CON groups and eight between the LAC and DMA groups, as detailed in Table S2.

Further analysis combining both VIP > 1 and *p*-value < 0.05 (illustrated in Figure 6A,B and detailed in Table S2) facilitated the identification of 16 characteristic metabolites in the comparison between the DMA and CON groups (highlighted on the left side of Figure 6C). These metabolites include increases in lactate and glucose and decreases in isoleucine, phenylalanine, leucine, tyrosine, valine, uridine, acetate, tryptophan, carnosine, methylsuccinate, taurine, proline, histidine, and aspartate. When comparing the LAC and DMA groups, eight characteristic metabolites were identified (shown on the right side of Figure 6C): six that were upregulated (tyrosine, acetate, leucine, isoleucine, phenylalanine, and valine) and two that were downregulated (lactate and glucose). Notably, these eight metabolites also appeared in the DMA vs. CON comparison, demonstrating inverse concentration trends when analyzed across the different group comparisons.

A Venn diagram (center in Figure 6C) visually summarizes the overlap, highlighting these eight characteristic metabolites shared between the two comparisons. This diagram effectively highlights their significant yet contrasting changes in concentration, reinforcing their potential role in differentiating metabolic responses between groups. This comprehensive analysis highlights the intricate metabolic changes associated with lactate treatment and DMA-induced muscle atrophy, providing a clearer understanding of the underlying mechanisms.

### 2.6. Lactate Altered Metabolic Pathways of Gastrocnemius Muscles of Suspended Mouse

**Figure 7:**
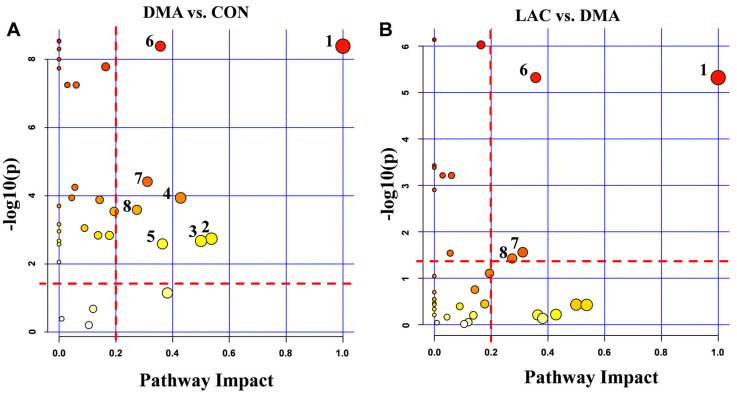
Significantly altered metabolic pathways in mouse gastrocnemius from pairwise comparisons. (**A**) Comparison between the DMA and CON groups revealed eight significantly altered pathways: 1. Phenylalanine, tyrosine, and tryptophan biosynthesis; 2. Alanine, aspartate, and glutamate metabolism; 3. D-glutamine and D-glutamate metabolism; 4. Taurine and hypotaurine metabolism; 5. Glutathione metabolism; 6. Phenylalanine metabolism; 7. Histidine metabolism; 8. Arginine and proline metabolism. (**B**) Comparison between the LAC and DMA groups identified four significantly altered pathways: 1. Phenylalanine, tyrosine, and tryptophan biosynthesis; 6. Phenylalanine metabolism; 7. Histidine metabolism; 8. Arginine and proline metabolism.

Metabolic pathway analysis was performed to identify significant pathways using PIV > 0.2 and *p*-value < 0.05 as criteria (Figure 7, Tables S3 and S4). This analysis revealed eight significant pathways affected in the comparison between the DMA and CON groups and four significant pathways affected in the comparison between the LAC and DMA groups. Four common significant pathways were identified in both comparisons: phenylalanine, tyrosine, and tryptophan biosynthesis; phenylalanine metabolism; histidine metabolism; and arginine and proline metabolism. These results highlight the critical influence of phenylalanine-related pathways in both the development of hindlimb suspension-induced muscle atrophy and its mitigation by lactate treatment. The significant pathways identified are intricately linked to amino acid metabolism, energy production, the mitigation of oxidative stress, and the replenishment of the tricarboxylic acid (TCA) cycle.

## 3. Discussion

Lactate plays a critical role in a wide range of physiological and pathological conditions [22]. This study elucidates the beneficial effects of lactate treatment on DMA-impaired skeletal muscle, a condition characterized by progressive deterioration and loss of muscle function due to prolonged inactivity. By using NMR-based metabolomic analysis, we investigated the metabolic changes in the gastrocnemius muscles of DMA mice compared to CON mice and evaluated the efficacy of lactate treatment in counteracting these adverse changes. 

**Figure 8:**
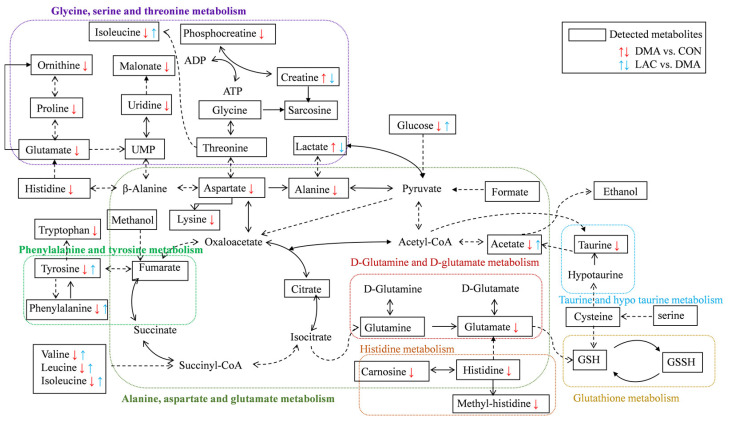
Illustration of lactate treatment improving impaired metabolic pathways and reversing the altered concentration trends of key metabolites in the gastrocnemius muscles of DMA mice compared to controls. A dotted-line arrow represents a multi-step reaction process, while a solid-line arrow represents a one-step reaction process. The following abbreviations are used: ATP stands for adenosine triphosphate, ADP for adenosine diphosphate, GSH for reduced glutathione, and GSSG for oxidized glutathione.

Our results show that lactate treatment significantly attenuates muscle mass loss and improves muscle functionality. This beneficial outcome is attributed to the downregulation of Murf1, a key marker of muscle atrophy; decreases in protein ubiquitination and hydrolysis; and the upregulation of MYHC expression. Importantly, lactate rescues perturbations in four significant pathways in the DMA gastrocnemius, namely phenylalanine, tyrosine, and tryptophan biosynthesis; phenylalanine metabolism; histidine metabolism; and arginine and proline metabolism. These pathways are closely related to the TCA cycle, amino acid metabolism, oxidative stress mitigation, and urea cycle metabolism, as shown in Figure 8. Notably, lactate also reverses the altered concentration trends of eight characteristic metabolites in the DMA gastrocnemius compared to the CON group. These results highlight the potential of lactate as an effective clinical strategy to combat the debilitating effects of prolonged inactivity, diminished exercise capacity, and muscle atrophy, particularly in conditions simulating microgravity.

### 3.1. Role of Lactate in Mitigating Disuse-Induced Muscle Atrophy and Improving Muscle Strength

Lactate treatment not only slightly reduced the body weight of LAC mice compared to DMA mice but also significantly increased the mass of their gastrocnemius muscles, a critical skeletal muscle component. The removal of epididymal fat highlighted the dual role of lactate in counteracting suspension-induced muscle atrophy and optimizing fat metabolism relative to body weight, which is consistent with previous research [23,24]. Furthermore, assessments of muscle tension in isolated gastrocnemius muscle from LAC mice revealed that lactate significantly increased peak muscle fiber contractility and endurance, underscoring its efficacy in alleviating DMA and enhancing the natural adaptation of skeletal muscle to disuse.

Extensive research has highlighted the key signaling pathways involved in muscle atrophy, particularly the downregulation of the PI3K/Akt/mTOR pathway, which leads to reduced muscle protein synthesis, the dephosphorylation of FOXO, and increased expression of proteolytic genes. Therapeutic strategies are often aimed at blocking the ubiquitin–proteasome pathway [25,26].

Our results indicate that lactate significantly reduces Murf-1 levels while increasing MYHC levels in the gastrocnemius muscle, suggesting the dual ability of lactate to increase muscle protein synthesis and prevent protein degradation. This dual action positions lactate as a promising agent for improving DMA outcomes. Lactate treatment appears to mitigate DMA by reducing Murf-1-linked protein degradation signals and counteracting the suppression of MYHC observed in suspension scenarios.

### 3.2. Role of Lactate in Regulating Branched-Chain Amino Acid Metabolism and Energy Metabolism

The gastrocnemius muscles of DMA mice showed a notable increase in glucose and lactate levels, indicating a disruption in energy metabolism. This suggests a shift towards glycolysis as the main source of ATP to protect against protein degradation.

DMA was associated with mitochondrial dysfunction and impaired oxidative phosphorylation, resulting in lactate accumulation due to the delayed entry of pyruvate into the TCA cycle [27]. Accumulated lactate is limited by blood circulation caused by hindlimb suspension, and the efficiency of diffusion into the liver is reduced. Lactate intervention increases its circulation flux, improves ATP synthesis efficiency, and effectively slows down muscle atrophy. However, this is only speculation. The existing research does not explain the changes in the levels of lactate and related enzymes in the liver and blood in the disuse muscle atrophy model, which is also a deficiency of our research work. In the future, we will consider collecting samples of liver and lactate as an important evidence supplement [28].

Furthermore, DMA was characterized by impaired amino acid metabolism, particularly a reduction in branched-chain amino acids (BCAAs) such as leucine, isoleucine, and valine. BCAAs, which are predominantly metabolized in skeletal muscle, comprise approximately 35% of the essential amino acids in muscle protein [29]. They are critical for stimulating protein synthesis, preventing hydrolysis, and serving as an energy substrate, especially leucine, which activates the mTOR pathway to increase energy metabolism and inhibit protein catabolism. The observed decrease in BCAAs suggests that muscle atrophy may be due to insufficient substrates for protein synthesis, hindering the ability to sustain the demands of protein synthesis. The reversal of BCAA downregulation by lactate treatment underscores the beneficial regulatory effects of lactate on DMA, primarily through the regulation of BCAA metabolism and energy metabolism.

### 3.3. Role of Lactate in Regulating Phenylalanine-Related Metabolic Pathways

Our analysis revealed that phenylalanine and tyrosine, two characteristic metabolites, were consistently identified in both the DMA vs. CON and LAC vs. DMA comparisons, with significantly reduced levels in the gastrocnemius muscles of DMA mice compared to controls. Notably, lactate treatment was able to partially correct these changes in metabolite concentrations. In addition, both pairwise comparisons highlighted two significant pathways: phenylalanine metabolism and phenylalanine, tyrosine, and tryptophan biosynthesis. These results suggest that DMA impairs phenylalanine-related pathways, while lactate treatment appears to ameliorate these impairments by affecting the significantly perturbed pathways.

In the future, we plan to further investigate phenylalanine-related pathways to uncover their essential contribution to the pathogenesis of disuse atrophy. By focusing on these pathways and examining the effects of interventions such as exercise and lactate supplementation, we aim to uncover key regulatory mechanisms within phenylalanine-related metabolic pathways. This approach is expected to identify potential therapeutic targets or novel drug candidates that could effectively counteract the detrimental effects of disuse atrophy.

### 3.4. Enhanced Recovery of DMA-Damaged Gastrocnemius Muscle via Amino Acid Supplementation

Our study found significant decreases in the levels of amino acids such as alanine, proline, lysine, histidine, and tryptophan in the gastrocnemius muscles of DMA mice, which is consistent with previous research showing decreases in these amino acids in various disease states [30,31,32,33,34]. Furthermore, our analysis revealed significant disruptions in three important metabolic pathways in DMA mice compared to controls: alanine, aspartate, and glutamate metabolism; histidine metabolism; and phenylalanine, tyrosine, and tryptophan biosynthesis. These findings suggest a potential connection to the impaired skeletal muscle condition observed in DMA mice and indicate that targeted supplementation of these amino acids may help improve muscle mass and functionality.

Moreover, the study found that taurine levels were significantly decreased in the DMA gastrocnemius muscle; taurine is known for its ability to strengthen muscles and act as an antioxidant. The research also showed a significant disturbance in taurine and hypotaurine metabolism. Previous studies have shown that taurine supplementation can help reduce muscle and nerve tissue damage caused by skeletal muscle atrophy by reducing the expression of the MuRF1 gene and caspase 3 in tissues [35]. These findings suggest that taurine may be an effective therapeutic agent against DMA-induced muscle wasting. Therefore, supplementing with strategic amino acids, including taurine, could be a promising approach to combat the negative effects of disuse-induced muscle atrophy. 

## 4. Materials and Methods

### 4.1. Animal Experiments

All procedures involving animals were carefully conducted according to the ethical standards approved by the Ethics Review Committee of Xiamen University (approval number: XMULAC20220200), ensuring strict adherence to guidelines for the humane treatment and welfare of laboratory animals. The living conditions of the mice were strictly controlled, with a 12 h light/dark cycle, a stable ambient temperature of 23 °C ± 3 °C, and a relative humidity of 70% ± 5%. After a one-week acclimatization period, the mice were systematically divided into three different groups: the control (CON) group, the disuse-induced muscle atrophy model (DMA) group, and the lactate-treated (LAC) group, each consisting of 12 mice. A comprehensive schematic of the animal experimental protocol is shown in Figure S1.

The DMA mice were subjected to the hindlimb suspension procedure to induce a state of disuse muscle atrophy, as described in previous studies [36]. This involved carefully suspending the mice by their tails using medical-grade cotton threads, adjusted to a height sufficient to prevent their hind legs from touching the ground, thereby simulating a microgravity environment like weightlessness. Throughout this period, all mice had unrestricted access to food and water.

The LAC mice received daily intraperitoneal injections of sodium lactate (#71718, Sigma–Aldrich, Shanghai, China) (1 g/kg/day) concurrent with the establishment of the DMA model. The dosage of lactate was selected based on its proven therapeutic efficacy in promoting cell proliferation, as has been established in previous studies and supported by literature indicating beneficial outcomes [15,18]. 

Food consumption and body weight was carefully monitored throughout the experiment. At the end of the six weeks, comprehensive assessments were conducted according to the protocols described in Section 4.2 and Section 4.3. These assessments focused on the skeletal muscle strength and functional abilities of the mice and included an assessment of the contractility of the gastrocnemius muscle and morphometric analysis of its muscle fibers. These evaluations provided critical insight into the effectiveness of lactate treatment in mitigating the detrimental effects of DMA on skeletal muscle.

### 4.2. Assessment of Gastrocnemius Muscle Contractility

After euthanasia, each gastrocnemius muscle was meticulously dissected and immediately immerged in a Petri dish containing Krebs solution, which was adjusted for physiological precision. The muscle was then carefully prepared for mechanical evaluation. One end of the muscle was attached to a precision transducer with surgical sutures, while the opposite end was connected to a fixed hook at the base of a chamber filled with a specially formulated physiological saline solution. This solution, consisting of 10 mM glucose, 2.5 mM CaCl_2_, 10 mM HEPES buffer, 140 mM NaCl, 5 mM KCl, and 2 mM MgCl_2_, was designed to mimic the natural ionic environment of the muscle, facilitating accurate assessment of contractility.

The time from induction of anesthesia to the start of mechanical muscle testing was consistently maintained at approximately three minutes for each mouse. During this interval, the hindlimb was prepared by excising the skin to expose the Achilles tendon, gastrocnemius, and soleus muscles. Electrodes were then attached to the gastrocnemius muscle to initiate the contractility testing. After evaluation, humane euthanasia was performed by cervical dislocation. The gastrocnemius muscle was immediately excised and quickly preserved in liquid nitrogen to ensure protocol uniformity and procedural consistency across all experimental groups.

After stabilization in this solution, the contractile properties of the muscle were rigorously analyzed, focusing on its peak contractile force (T_max_) and endurance capacity. Muscle endurance was quantified by the time required for T_max_ to decrease to half of its initial value [37].

These critical metrics were accurately measured using the BL-420F Biosignal Acquisition and Analysis System (Chengdu Taimeng Software Co., Ltd., Chengdu, China). By adopting this methodological framework, a thorough evaluation of the functional integrity and resilience of the gastrocnemius muscle became possible within the scope of this study.

### 4.3. Morphometric Evaluation of Gastrocnemius Muscle Fibers

Gastrocnemius muscle samples were initially fixed in 4% paraformaldehyde and subjected to a 24 h fixation period to preserve their structural integrity. After fixation, the samples underwent a rigorous dehydration process through a graded ethanol series and were finally embedded in a paraffin matrix to allow accurate sectioning. From these embedded tissues, 5 μm-thick sections were meticulously prepared and then stained with hematoxylin and eosin (H&E). This traditional histologic staining technique distinguishes between cellular and extracellular matrix components, significantly improving the delineation of muscle fiber architecture.

Following staining, the sections were carefully mounted and sealed with coverslips to protect their morphological integrity. High-resolution images of these stained sections were captured using a sophisticated light microscope equipped with digital imaging technology. These images provided the basis for the subsequent quantitative morphometric analysis.

The cross-sectional area and diameter of individual muscle fibers were determined using ImageJ software (version 1.51j8), a respected image processing tool known for its precision in morphometric evaluations. This digital examination allowed for precise measurement of muscle fiber dimensions and offered a crucial understanding of the morphological alterations in the gastrocnemius muscle under various experimental conditions.

### 4.4. Preparation of Gastrocnemius Muscle Samples for Western Blot Analysis

For protein expression analysis, mouse gastrocnemius muscle tissues were first homogenized in a strong RIPA lysis buffer (Solarbio Technology Co., Ltd., Beijing, China) supplemented with protease and phosphatase inhibitors to inhibit enzymatic degradation of proteins. This homogenization was performed under refrigerated conditions to maintain protein stability. Tissue lysates were then disrupted by sonication for one minute to achieve thorough cell lysis and optimal release of intracellular proteins.

After disruption, the lysates were centrifuged at 12,000× *g* for 10 min at 4 °C. This critical step facilitated the separation of soluble proteins in the supernatant from the cellular debris. The transparent supernatant containing the isolated proteins was then carefully decanted into a fresh tube for subsequent assay.

Protein concentrations in the supernatant were accurately determined using the BCA Protein Assay Kit (Lab Lead, Xiamen, China). Based on these measurements, samples were normalized by incorporating an SDS-PAGE loading buffer to equalize protein concentrations across samples to ensure consistency for comparative analysis.

Before electrophoresis, samples were denatured by incubation at 95 °C for 10 min in a thermal block. This critical step was designed to unfold the proteins, breaking down their tertiary and quaternary structures to bring them into a linear configuration suitable for gel electrophoresis. The prepared samples were then either processed immediately for further experimental steps or stored safely at −80 °C for later analysis.

### 4.5. Western Blot Analysis and Protein Expression Evaluation

The Western blotting was meticulously performed according to a strictly defined protocol. This process began with the resolution of protein samples by SDS-PAGE, using gels with a gradient of 8% to 15% to skillfully separate proteins across a spectrum of molecular weights. Following electrophoresis, the proteins were uniformly transferred to PVDF membranes (GE Healthcare, Shanghai, China) using the WIX transfer system for efficient translocation. The PVDF membrane was then immersed in a blocking solution of 5% nonfat dry milk and gently agitated at 80 rpm for one hour to achieve thorough coverage and effective blocking. The membrane was then washed several times with TBST to remove any residual blocking solution before being incubated with the specific primary antibody overnight for 8 to 12 h under optimal conditions, including MYHC (sc-376157, Santa Cruz Biotechnology, Dallas, TX, USA), Murf1 (MP3401, ECM bioscience, Aurora, CO, USA), and GAPDH (10494-1-AP, Proteintech, Wuhan, China). After the primary antibody incubation, the membrane was washed three times with TBST for 5 to 15 min each and then incubated with a horseradish peroxidase-conjugated secondary antibody for 1 h at room temperature (Cell Signaling Technology, Danvers, MA, USA).

For signal visualization, the membrane was treated with an enhanced chemiluminescence reagent (ECL, Beyotime Biotechnology, Shanghai, China) and then placed in a chemiluminescence detection device maintained at 4 °C to refine signal detection conditions and ensure the integrity of the visualized protein bands. The resulting protein bands were then subjected to densitometric analysis using ImageJ software to facilitate accurate quantification.

### 4.6. Preparation of Gastrocnemius Muscle Samples for NMR Experiments

The preparation of gastrocnemius muscle samples for NMR spectroscopic analysis was performed meticulously according to well-established protocols cited in the literature [38,39]. Upon completion of the animal experiments, the mice were first anesthetized with phenobarbital and then humanely euthanized by cervical dislocation. Immediately after euthanasia, the skin of the hindlimbs was gently peeled back to expose the Achilles, gastrocnemius, and soleus muscles. These muscles were quickly excised from the limbs and placed in sterile 1.5 mL tubes. To immediately halt tissue metabolism and inhibit enzymatic activity, the dissected samples were immersed in liquid nitrogen within seconds of dissection to ensure the preservation of their biochemical integrity for subsequent NMR analysis.

To homogenize the tissue samples, approximately 100 mg of gastrocnemius muscle was weighed and transferred into a homogenization tube. Pre-cooled methanol and water at a ratio of 1:0.95 (*w*/*v* = 1:10) were added to the tube along with grinding steel beads. The sample was then homogenized using a 65 Hz grinder at a temperature of 4 °C for 60 s. After this initial grinding, pre-cooled chloroform was added at a ratio of *w*/*v* = 1:10, and the homogenate was processed again under the same conditions. The primary functions of methanol and chloroform were to quench protein activity and facilitate the extraction of water-soluble and lipid-soluble metabolites, respectively. Our focus was on water-soluble metabolites; therefore, after homogenization, the mixture was centrifuged to separate the phases, and only the aqueous phase was retained for further analysis. After the methanol was evaporated, any remaining water was removed by lyophilization, resulting in a dry powder of the extracted metabolites.

This concentrated metabolite powder was then dissolved in 600 μL of an NMR-compatible buffer containing 50 mM phosphate buffer with 0.05 mM TSP (3-(trimethylsilyl) propionic-2,2,3,3-d4-acid) for accurate chemical shift referencing. The solution was adjusted to pH 7.4 and prepared in deuterium oxide (D_2_O) to provide a deuterium lock signal, which is essential for NMR detection. After centrifugation at 12,000× *g* to ensure a clear solution, the transparent supernatant was carefully transferred to a 5 mm NMR tube, ready for subsequent NMR spectroscopic examination.

### 4.7. NMR Experiments

All NMR experiments were performed at 298 K using a high-resolution Bruker Avance III HD 850 MHz spectrometer equipped with a TCI cryogenic probe for enhanced detection sensitivity. One-dimensional (1D) ^1^H NMR spectra were recorded using the standard NOESYGPPR1D pulse sequence, designated [RD-G1-90°-τm-G2-90°-ACQ], which was specifically designed for effective water suppression. This was achieved by suppressing the water signal during both the relaxation delay (RD) and the mixing time (τm), with the inclusion of pulse gradients G1 and G2 to further enhance the attenuation of the water peak. The main experimental parameters for the 1D ^1^H NMR spectra were as follows: τm = 10 ms; ACQ = 2.66 s; RD = 4 s. A total of 64 transients were collected into 64 K data points, with a spectral width of 20 ppm.

Additionally, two-dimensional (2D) ^1^H-^13^C heteronuclear single quantum coherence (HSQC) spectra were recorded to confirm the resonance assignments of the metabolites using the hsqcetgpprsisp2.2 pulse sequence, providing detailed insight into the carbon–hydrogen connectivity within the metabolites.

### 4.8. NMR Spectral Data Processing and Resonance Assignment

The NMR spectral data underwent initial preprocessing and Fourier transformation using Topspin software (version 4.4.0). This was followed by extensive spectral refinement in MestReNova 9.0 software, including phase correction, baseline adjustment, and chemical shift calibration to ensure accuracy and consistency across all spectra. The chemical shift calibration was carefully anchored to the methyl resonance peak of TSP (δ 0.00 ppm). The water resonance region (4.75–4.85 ppm) was excluded to eliminate any confounding effects of the water signal on the analysis. In addition, the data were normalized relative to the TSP signal and the mass of the gastrocnemius muscle tissue to ensure that the resulting data matrix accurately reflected the relative metabolite concentrations, thereby facilitating robust and meaningful multivariate analysis.

The processed spectra were subsequently overlaid to enable a comprehensive analysis. This overlay facilitated a meticulous peak alignment process on the combined spectra, guaranteeing consistency in peak positions. The aligned spectra were then segmented into discrete regions with a spectral region (bin) of 0.001 ppm. These quantified data were imported into MATLAB R2015b, where they were organized into a structured data matrix, laying the groundwork for in-depth multivariate statistical analysis.

Resonance assignments of metabolites were conducted based on the 1D ^1^H spectra using a combination of the Chenomx NMR Suite software (version 8.3, Chenomx Inc., Edmonton, AB, Canada), the Human Metabolome Database (HMDB, http://www.hmdb.ca/, accessed on 5 March 2023), and the relevant literature. These assignments were further confirmed by using 2D ^1^H-^13^C HSQC spectra.

### 4.9. Multivariate Statistical Analysis and Significant Metabolite Identification

The NMR dataset was subjected to multivariate statistical analysis using MetaboAnalyst 5.0 (http://www.MetaboAnalyst.ca, accessed on 13 March 2023) and SIMCA-P+ software (version 14.1) [40]. Pareto scaling was applied to the initial data to effectively reduce the influence of varying metabolite concentrations on analytical results. Unsupervised principal component analysis (PCA) was used to examine the metabolic patterns between the groups, elucidate potential outliers, and delineate group differences. Supervised partial least squares discriminant analysis (PLS-DA) was then performed to improve discrimination among the different metabolic profiles of the groups. The robustness of the PLS-DA model was rigorously evaluated by calculating the explanatory power (R^2^Y (cum)) and predictive accuracy (Q^2^Y (cum)) of the model using a 200-iteration permutation test. These parameters, with values close to one indicating optimal model performance, serve as indicators of the model’s ability to accurately explain and predict the variance in the dataset. 

The PLS-DA model was used to identify significant metabolites based on a criterion of variable importance in projection (VIP) > 1. These metabolites were considered important contributors to the observed metabolic differences between groups and therefore merited further investigation for their potential biological relevance.

### 4.10. Univariate Statistical Analysis and Differential Metabolite Identification

Metabolite concentration data were subjected to univariate statistical analysis using SPSS software (version 23.0). Pairwise comparisons between groups were performed using one-way analysis of variance (ANOVA), followed by Tukey’s multiple comparison test to detect statistically significant differences. Differential metabolites between the groups were identified using a criterion of *p* < 0.05. Characteristic metabolites were identified using the dual criteria of *p* < 0.05 and VIP > 1. This approach facilitated the identification of metabolites that were both statistically significant and highly influential in the model, thereby highlighting those most relevant to the observed metabolic differences among the groups.

### 4.11. Metabolic Pathway Analysis and Significant Pathway Identification

Metabolite concentration data were analyzed using the pathway analysis module of MetaboAnalyst 5.0 (https://www.metaboanalyst.ca, accessed on 15 March 2023)) to uncover significant pathways affected by changes in metabolite levels. A dual approach was used for this analysis. First, metabolite set enrichment analysis identified significant pathways where *p*-value < 0.05, indicating pathways enriched in metabolites that varied significantly between samples, and suggesting their biological relevance. Subsequently, a pathway topological analysis assigned a pathway impact value (PIV), where pathways for which PIV > 0.2 were considered significant. This highlighted the essential roles and interactions of metabolites within these pathways, indicating not only statistical significance but also deep biological importance. Pathways that met both criteria (*p*-value < 0.05 and PIV > 0.2) were considered biologically significant and potentially responsive to experimental conditions.

### 4.12. Overview of Statistical Analyses

Experimental data were presented as mean ± SD, and statistical analysis was performed using IBM SPSS Statistics 22.0 software (IBM, New York, NY, USA). One-way ANOVA followed by Tukey’s multiple comparison test was used to compare the three groups of mouse gastrocnemius. Statistical significance was determined as *p* > 0.05 (no statistical significance, ns), *p* < 0.05 (*), *p* < 0.01 (**), *p* < 0.001 (***), *p* < 0.0001 (****).

## 5. Conclusions

Our study demonstrated that lactate treatment effectively addresses DMA in the mouse model. Lactate significantly preserves skeletal muscle mass and function, primarily by suppressing the expression of Murf1. This protective effect is attributed to decreased protein degradation and increased MYHC expression, underscoring the ability of lactate to enhance muscle resilience. In addition, lactate significantly affects four key pathways in the DMA gastrocnemius: the biosynthesis of phenylalanine, tyrosine, and tryptophan; phenylalanine metabolism; histidine metabolism; and arginine and proline metabolism. These pathways are integral to the TCA cycle, amino acid metabolism, the oxidative stress response, and urea cycle metabolism, demonstrating the broad metabolic impact of lactate. Specifically, lactate plays crucial roles in regulating branched-chain amino acid metabolism and energy metabolism, as well as phenylalanine-related pathways. Notably, lactate also normalized the levels of eight essential metabolites in DMA mice, including isoleucine, phenylalanine, leucine, tyrosine, valine, lactate, acetate, and glucose, compared to control mice. These changes underscore the therapeutic promise of lactate in mitigating the negative consequences of prolonged inactivity, reduced exercise capacity, and muscle atrophy. Our results support the strategic use of lactate in clinical approaches to treat muscle-wasting disorders, providing a novel therapeutic pathway that exploits the multiple benefits of lactate. This study not only highlights the importance of lactate in maintaining muscle health but also paves the way for further investigation into its broader applications in muscle recovery and rehabilitation. 

Although NMR spectroscopy offers excellent reproducibility and allows absolute quantification, making it highly suitable for cross-study comparisons, it also has certain limitations [41]. Despite the use of a high-field 850 MHz NMR spectrometer with a TCI cryogenic probe, which significantly increases detection sensitivity, the range of tissue metabolites that can be identified remains limited due to the inherently lower sensitivity and resolution of NMR compared to other techniques. This limitation has impacted the breadth of metabolic pathways that can be analyzed and the depth of coverage that can be achieved. To address these limitations and enrich the metabolic insights from our study, future work will incorporate LC-MS-based metabolomics. This approach will be applied to a broader range of biological samples, including skeletal muscle, liver tissue, and serum from the DMA model. By integrating LC-MS, we aim to provide a more comprehensive and systematic assessment of the effects of lactate treatments and to elucidate the underlying metabolic mechanisms, particularly those related to key metabolic cycles such as the TCA cycle, the Cori cycle, and the Cahill cycle.

## Data Availability

The data presented in this study are available on request from the corresponding author.

## Supplementary Materials

The following supporting information can be downloaded at: https://www.mdpi.com/article/10.3390/molecules29102216/s1, Figure S1: Design scheme for the animal experiment; Figure S2: Representative 2D ^1^H-^13^C HSQC spectra of aqueous metabolites from mouse gastrocnemius muscle; Figure S3: Permutation test of the PLS-DA model for the 1D ^1^H-NMR spectra recorded from mouse gastrocnemius muscles (*n* = 200). Figure S4: Heatmap illustrating the variation in metabolite levels between groups; Table S1: Resonance assignments of aqueous metabolites in 1D ^1^H-NMR spectra from mouse gastrocnemius muscles; Table S2. Univariate statistical analysis of relative integrals of metabolites in the CON, DMA, and LAC groups of mouse gastrocnemius; Table S3. Significantly perturbed metabolic pathways in the DMA gastrocnemius compared to controls; Table S4. Significantly perturbed metabolic pathways in the LAC gastrocnemius compared to the DMA gastrocnemius.
