# Supplementary material for: Deciphering the Therapeutic Role of Lactate in Combating Disuse-Induced Muscle Atrophy: An NMR-Based Metabolomic Study in Mice
Source: Molecules. 2024 May 9;29(10):2216. doi: 10.3390/molecules29102216 (PMC11124173; doi:10.3390/molecules29102216)
Supplement: Supplementary file 1 [file molecules-29-02216-s001.zip › molecules-2966248-supplementary.pdf]

Supplementary Materials

Figure S1

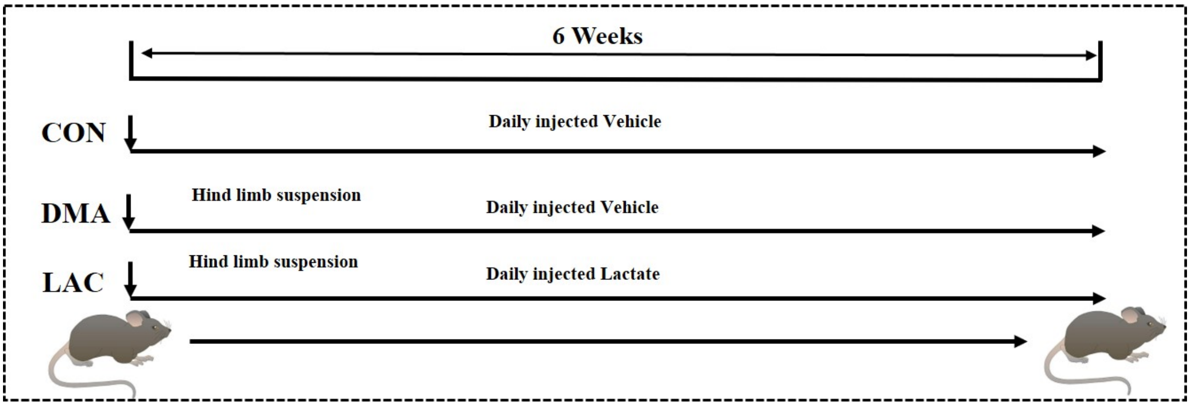

Figure S1. Design scheme for the animal experiment.

Figure S2

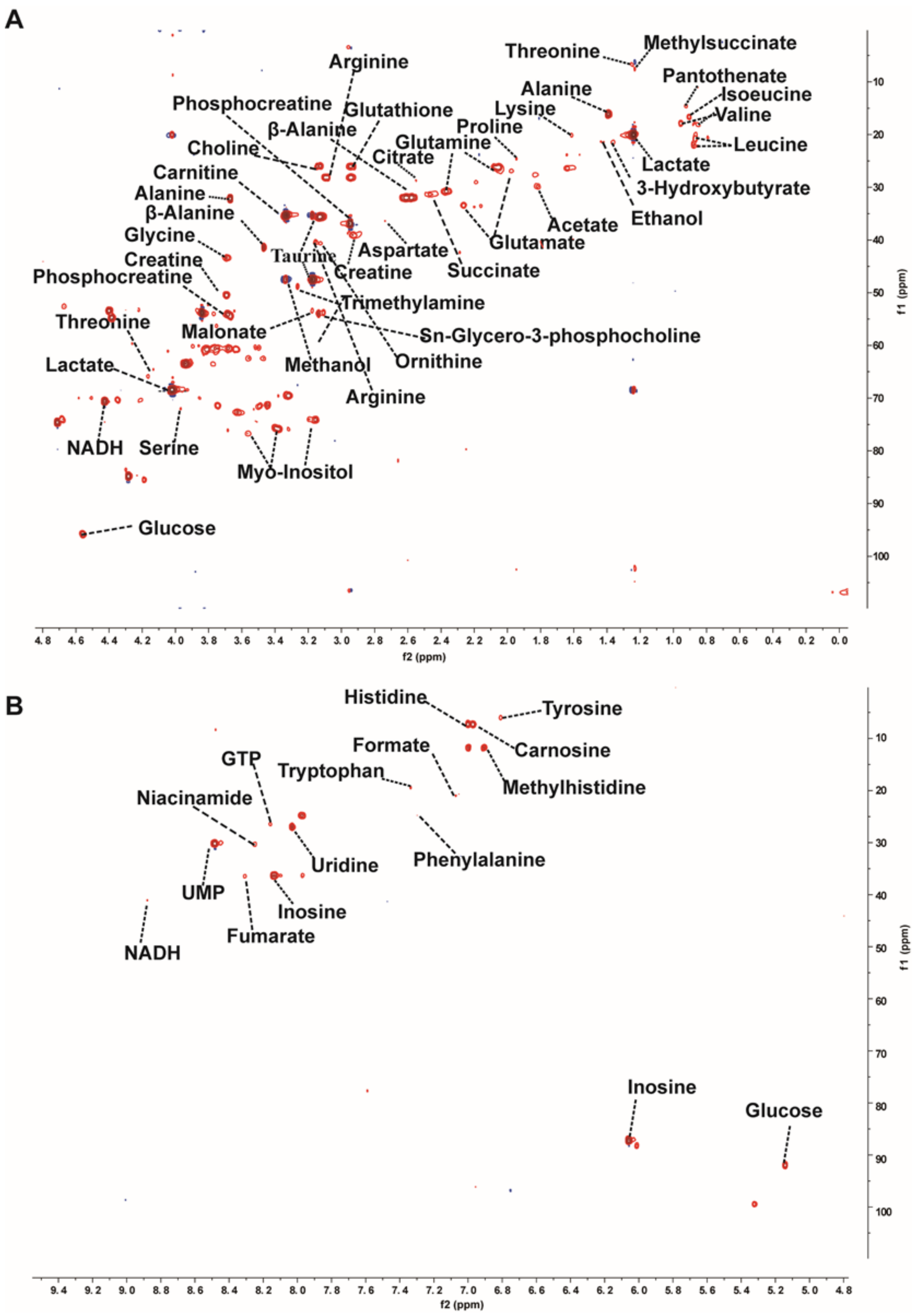

Figure S2. Representative 2D ^{1}H-^{13}C HSQC spectra of aqueous metabolites from mouse gastrocnemius muscles.

Figure S3

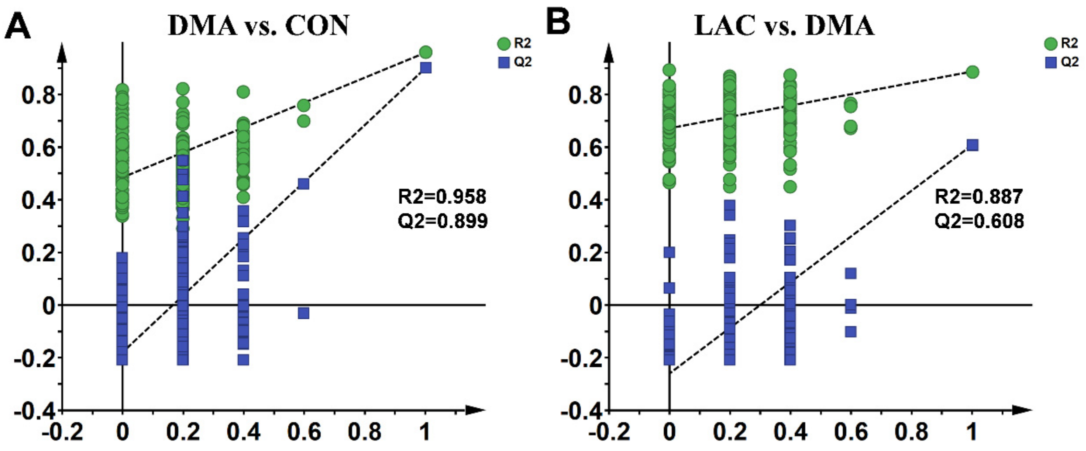

Figure S3. Permutation test of the PLS-DA model for the 1D 1H-NMR spectra recorded from mouse gastrocnemius muscles (n=200). (A) DMA vs. CON ($R^2Y(cum)$ = 0.958, $Q^2Y(cum)$ = 0.899), (B)LAC vs. DMA (R^2 Y(cum) = 0.887, Q^2 Y(cum) = 0.608).

Figure S4

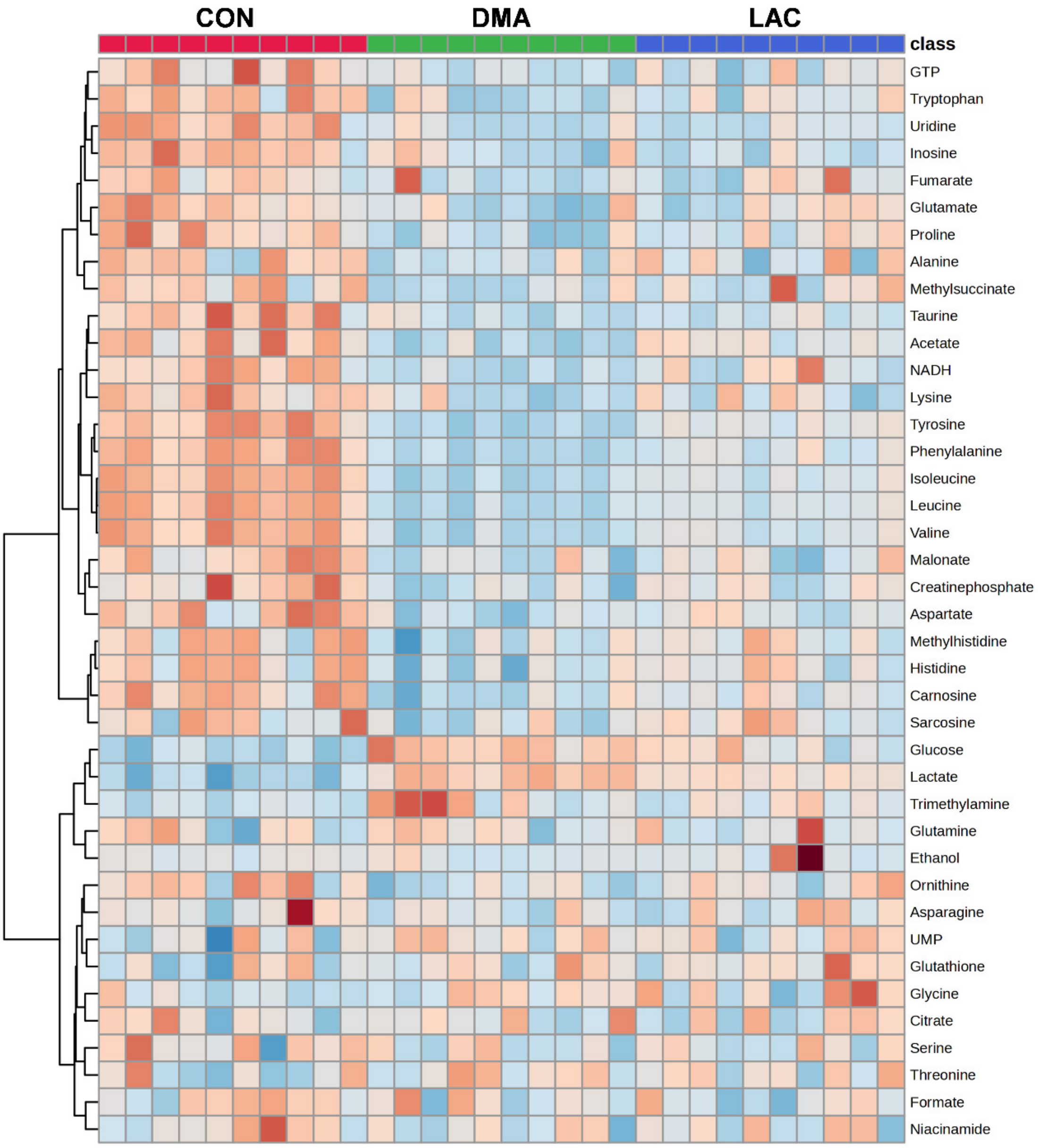

Figure S4. Heatmap illustrating the variation in metabolite levels between groups.

Table S1. Resonance assignments of aqueous metabolites in 1D ^{1}H NMR spectra from mouse gastrocnemius muscles.

No.	Metabolite	Chemical shift (ppm) (Multiplicity)	Assignment
1	Leucine	0.96(d), 0.97(d), 1.69(m), 1.70(m), 1.73(m), 3.73(m)	α-CH_3, α-CH_3, γ-CH, α-CH_3, β-CH_2, α-CH
2	Isoleucine	0.94(t), 1.01(d), 1.21(m), 1.42(m), 2.00(m), 3.67(d)	δ-CH_3, γ-CH_3, half γ-CH_2, half γ-CH_2, β-CH, α-CH
3	Valine	0.99(d), 1.05(d), 2.26(m), 3.60(d)	γ-CH_3, γ-CH_3, β-CH, α-CH
4	Methyl succinate	1.08(d)	α-CH_3
5	Ethanol	1.18(t), 3.65(q)	CH_3, CH_2
6	Lactate	1.32(d), 4.11(q)	β-CH_3, α-CH
7	Alanine	1.46(d), 3.76(q)	β-CH_3, α-CH
8	Acetate	1.91(s)	β-CH_3
9	Proline	3.33(m)	half δ-CH_2
10	Glutamate	2.09(m), 2.35(m)	β-CH_3, half γ-CH_2
11	Citrate	2.52(d), 2.68(d)	half CH_2, half CH_2
12	Carnosine	2.67(q),3.10(m),3.22(t),4.47(q),7.05(s),8.02(s)	CH_2, N-CH, CH, N-CH=N
13	Sarcosine	2.72(s),3.61(s)	CH_3, α-CH_2
14	Aspartate	2.68(dd), 2.81(dd), 3.90(dd)	β-CH_2, α-CH
15	Asparagine	2.84(q),2.93(q)	β-CH_2
16	Trimethylamine	2.88(s)	CH_3
17	Glutathione	2.15(m), 2.55(m), 2.96(m), 3.77(m), 4.56(m)	β-CH_2, γ-CH_2, CH_2-SH, α-CH&CH_2-NH, CH-NH
18	Arginine	1.68(m), 1.90(m), 3.24(t)	α-CH_2, β-CH_2, δCH_2
19	Lysine	1.70(m), 1.88(m), 3.03(t)	γ-CH_2, β-CH, εCH_2
20	Phosphate Creatine	3.05(s), 4.05(s)	N-CH_3, CH_2
21	Ornithine	3.04(m)	NH_2
22	Malonate	3.12(s)	α-CH_2

23	Taurine	3.25(t), 3.42(t)	α-CH_2, β-CH_2
24	Glycine	3.55(s)	CH_2
25	Methanol	3.36(s)	CH_3
26	Threonine	1.31(d), 3.59(d), 4.25(m)	γ-CH_2, β-CH
27	Glutamine	2.13(m), 2.45(m), 3.77(t)	γ-CH_2, β-CH_2, α-CH
28	Serine	3.83(q),3.95(q),3.98(q)	α-CH, β-CH_2
29	Niacinamide	7.60(dd),8.23(dd),8.70(dd),8.92(s)	α-CH, β-CH, N=CH, N-CH
30	Inosine	3.83(d),3.84(d),6.1(d),8.23(s),8.35(s)	CH, CH, N-CH=N, N-CH'=N
31	Tryptophan	7.19(t),7.27(t),7.32(s)	CH, CH, CH,
32	Phenylalanine	7.31(m), 7.35(m), 7.40(m)	2,6-CH, 3,5-CH, 4-CH
33	Histidine	7.06(s), 7.85(s)	5-CH,2-CH
34	Tyrosine	6.87(d), 7.17(d)	CH, CH
35	Methyl histidine	7.03(s), 7.73(s)	4-CH, 2-CH
36	Fumarate	6.51(s)	CH
37	Glucose	3.52(d), 3.71(t), 3.82(m), 5.23(d)	2-CH, 3-CH, 5,6-CH, 1-CH
38	UMP	3.40(m),4.26(m),4.35(m),4.41(m),5.99(m),8.11(m)	N-CH, CH, CH', C-P, N=C-O, N-CH
39	GTP	5.92 (d), 8.1 (s)	CH, CH
40	Uridine	5.90(d),7.86(d)	CH
41	NADH	6.03(d),6.08(s),8.16(s),8.20(m), 8.41(s),8.82(d),9.13(d),9.31(s)	NH_2, NH_2(CO), CH, β-CH, γ-CH, α-CH

Abbreviations: s, singlet; d, doublet; dd, double doublet; t, triplet; q, quartet; m, multiple; UMP, uridylic acid; GTP, guanosine triphosphate; NADH, nicotinamide adenine dinucleotide.

Table S2. Univariate statistical analysis of relative integrals of metabolites in the CON, DMA, and LAC groups of mouse gastrocnemius.

**Metabolites**	**mean ± SD**			**DMA *vs.* CON**	**LAC *vs.* DMA**	**ANOVA**	
	**CON**	**DMA**	**LAC**			**F**	**P**
Leucine	0.608 ±0.050	0.404 ±0.035	0.461 ±0.022	↓ ***	↑ **	79.739	<0.001
Isoleucine	0.260 ±0.019	0.172 ±0.015	0.198 ±0.012	↓ ***	↑ **	85.889	<0.001
Valine	0.136±0.014	0.084±0.011	0.100±0.007	↓ ***	↑ **	61.508	<0.001
Methylsuccinate	0.002±0.001	0.001±0.001	0.001±0.001	↓ *	ns	5.354	0.110
Ethanol	0.038±0.008	0.034±0.016	0.060±0.066	ns	ns	1.296	0.290
Lactate	18.031±1.388	22.755±0.96	21.374±0.436	↑ ***	↓ **	58.263	<0.001
Alanine	1.447±0.102	1.333±0.07	1.376±0.123	↓ *	ns	3.314	0.052
Acetate	0.126±0.017	0.089±0.009	0.108±0.008	↓ ***	↑ ***	23.498	<0.001
Proline	0.639±0.077	0.474±0.069	0.546±0.064	↓ **	ns	13.958	<0.001
Glutamate	0.355±0.039	0.274±0.053	0.303±0.045	↓ **	ns	7.861	0.002
Citrate	0.019±0.005	0.019±0.004	0.019±0.005	ns	ns	0.019	0.981
Carnosine	1.370±0.068	1.209±0.062	1.264±0.044	↓ **	ns	19.261	<0.001
Sarcosine	0.046±0.003	0.043±0.002	0.045±0.002	ns	ns	3.788	0.035
Aspartate	0.064±0.011	0.046±0.007	0.051±0.006	↓ **	ns	13.304	<0.001
Asparagine	0.014±0.005	0.012±0.002	0.013±0.004	ns	ns	0.769	0.473
Trimethylamine	0.009±0.002	0.019±0.01	0.013±0.004	↑ *	ns	5.628	0.009
Glutathione	0.053±0.016	0.059±0.011	0.062±0.012	ns	ns	1.121	0.340
Lysine	0.246±0.036	0.17±0.037	0.191±0.047	↓ **	ns	9.379	0.001
PhosphateCreatine	8.124±0.369	7.53±0.253	7.748±0.204	↓ **	ns	11.177	<0.001
Ornithine	0.024±0.007	0.017±0.005	0.021±0.005	↓ **	ns	4.885	0.015
Malonate	0.064±0.003	0.060±0.002	0.061±0.003	↓ **	ns	8.787	0.001
Taurine	10.899±0.701	9.667±0.374	9.742±0.258	↓ **	ns	20.461	<0.001
Glycine	0.615±0.064	0.673±0.071	0.693±0.145	ns	ns	1.646	0.212
Threonine	0.108±0.031	0.121±0.022	0.117±0.023	ns	ns	0.657	0.526
Glutamine	0.286±0.052	0.294±0.035	0.293±0.049	ns	ns	0.088	0.915

Serine	0.130±0.015	0.121±0.011	0.124±0.010	ns	ns	1.344	0.278
Niacinamide	0.030±0.002	0.029±0.001	0.029±0.001	ns	ns	1.146	0.333
Inosine	0.074±0.009	0.059±0.011	0.055±0.006	↓ **	ns	13.084	<0.001
Tryptophan	0.006±0.001	0.004±0.001	0.005±0.001	↓ **	ns	13.416	<0.001
Phenylalanine	0.063±0.006	0.042±0.003	0.048±0.004	↓ ***	↑ **	64.746	<0.001
Histidine	0.745±0.05	0.661±0.049	0.703±0.039	↓ **	ns	8.390	0.001
Tyrosine	0.066±0.007	0.043±0.003	0.053±0.003	↓ ***	↑ ***	61.561	<0.001
Methylhistidine	0.749±0.051	0.677±0.043	0.715±0.034	↓ **	ns	6.846	0.004
Fumarate	0.015±0.003	0.012±0.004	0.013±0.004	ns	ns	1.966	0.160
Glucose	0.106±0.012	0.157±0.014	0.134±0.018	↑ ***	↓ **	28.127	<0.001
UMP	0.023±0.002	0.025±0.001	0.024±0.001	ns	ns	1.398	0.264
GTP	0.014±0.001	0.010±0.001	0.010±0.001	↓ **	ns	8.917	0.001
Uridine	0.02±0.003	0.014±0.002	0.014±0.001	↓ **	ns	26.602	<0.001
NADH	0.002±0.001	0.003±0.001	0.002±0.001	ns	ns	12.204	0.283
Formate	0.004±0.001	0.005±0.001	0.004±0.001	ns	ns	1.147	0.330

Table S3. Significantly perturbed metabolic pathways in the DMA gastrocnemius compared to controls.

No	Metabolic Pathway	hit score	*p*-value	PIV
1	Phenylalanine, tyrosine and tryptophan biosynthesis	2/4	<0.0001	1
2	Alanine, aspartate and glutamate metabolism	7/28	0.0018	0.537
3	D-glutamine and D-glutamate metabolism	2/6	0.0021	0.500
4	Taurine and hypo taurine metabolism	1/8	0.0001	0.428
5	Glutathione metabolism	4/28	0.0025	0.364
6	Phenylalanine metabolism	2/12	<0.0001	0.357
7	Histidine metabolism	5/16	<0.0001	0.311
8	Arginine and proline metabolism	4/38	0.0002	0.274

Note: Metabolic pathways are ranked according to their PIVs and the highest PIV pathways are listed.

Table S4. Significantly perturbed metabolic pathways in the LAC gastrocnemius compared to the DMA gastrocnemius.

No	Metabolic Pathway	Hit score	*p*-value	PIV
1	Phenylalanine, tyrosine and tryptophan biosynthesis	2/4	<0.0001	1
2	Phenylalanine metabolism	2/12	<0.0001	0.357
3	Histidine metabolism	5/16	0.0276	0.311
4	Arginine and proline metabolism	4/38	0.0375	0.274

Note: Metabolic pathways are ranked according to their PIVs and the highest PIV pathways are listed first
